# The Impact of N_2_-Assisted High-Pressure Processing on the Microorganisms and Quality Indices of Fresh-Cut Bell Peppers

**DOI:** 10.3390/foods10030508

**Published:** 2021-02-28

**Authors:** Fan Zhang, Jingjing Chai, Liang Zhao, Yongtao Wang, Xiaojun Liao

**Affiliations:** 1 College of Food Science and Nutritional Engineering, China Agricultural University, Beijing 100085, China; bs20183060546@cau.edu.cn (F.Z.); s20183060850@cau.edu.cn (J.C.); zhaoliang1987@cau.edu.cn (L.Z.); liaoxjun@cau.edu.cn (X.L.); 2 National Engineering Research Center for Fruit & Vegetable Processing, Beijing Key Laboratory for Food Nonthermal Processing, Key Laboratory of Fruit and Vegetable Processing, Ministry of Agriculture, Beijing 100085, China

**Keywords:** bell peppers, high-pressure processing, fresh-cut bell peppers, nitrogen-assisted, storage quality, structural damage

## Abstract

This work aimed to evaluate the effects of N_2_-assisted high-pressure processing (HPP, 400 MPa/7.5 min and 500 MPa/7.5 min) on the microorganisms and physicochemical, nutritional, and sensory characteristics of fresh-cut bell peppers (FCBP) during 25 days of storage at 4 °C. Yeasts and molds were not detected, and the counts of total aerobic bacteria were less than 4 log_10_ CFU/g during storage at 4 °C. The total soluble solids and L* values were maintained in HPP-treated FCBP during storage. After the HPP treatment, an 18.7–21.9% weight loss ratio and 54–60% loss of hardness were found, and the polyphenol oxidase (PPO) activity was significantly inactivated (33.87–55.91% of its original activity). During storage, the weight loss ratio and PPO activity of the samples increased significantly, but the hardness of 500 MPa/7.5 min for treated FCBP showed no significant change (9.79–11.54 N). HPP also effectively improved the total phenol content and antioxidant capacity of FCBP to 106.69–108.79 mg GAE/100 g and 5.76–6.55 mmol Trolox/L; however, a non-negligible reduction in total phenols, ascorbic acid, and antioxidant capacity was found during storage. Overall, HPP treatments did not negatively impact the acceptability of all sensory attributes during storage, especially after the 500 MPa/7.5 min treatment. Therefore, N_2_-assisted HPP processing is a good choice for the preservation of FCBP.

## 1. Introduction

Bell peppers (or sweet peppers), a cultivar group of the species *Capsicum annuum* L., are characterized by their blocky shape, attractive color, and mild taste. Bell peppers contain extremely high levels of the antioxidants vitamin C and E. Furthermore, they contain moderate to high amounts of phenolics, flavonoids, and carotenoids, and capsanthin, lutein, and cryptoflavin have been found in peppers [1,2]. The consumption of these bioactive compounds provides the human body with protection against oxidative damage, thus reducing the incidence of degenerative diseases. Currently, the fresh market consumption of bell peppers is becoming increasingly popular, mainly attributed to their availability in a wide variety of shapes, sizes, colors, and distinctive flavors [3].

For the convenience of food preparation processes in restaurants and fast-food industries worldwide and personal consumption, numerous fruits and vegetables are packaged according to the process of fresh cutting [4]. Compared with fresh vegetables, fresh-cut vegetables are ready to eat without further treatment and are still in their fresh state, keeping all the advantages of fresh vegetables (e.g., color, shape, and nutrition). The market demand for fresh-cut vegetables has grown rapidly because of their health benefits and convenience. However, the short shelf life of fresh-cut vegetables is an undesirable problem, which needs to be addressed [5]. Respiration is well known as an important metabolic process, which causes the deterioration of fruits and vegetables both after harvest and during food processing, and throughout the storage time. Hence, in order to reduce the respiration rate and maintain the quality of vegetables, lowing the temperature of storage (i.e., cold storage and cold-chain) and decreasing the O_2_ level in packaged vegetables are recommended [6]. Additionally, N_2_ is widely used in packaged food processing, thereby helping to isolate O_2_ and retard oxidative processes and the growth of aerobic spoilage microorganisms [7]. Overall, the modification of the gaseous composition in food packaging has been demonstrated to reduce the physical–chemical deterioration and prolong the shelf life of packaged food.

As a successfully commercialized non-thermal technology, high-pressure processing (HPP) achieves a remarkably good sterilization effect and ensures that the original flavor and nutritional value of packaged products are retained [8,9]. Compared with traditional sterilization, HPP technology is less time-consuming and can maintain the nutritional value and delicate sensory properties of fruits and vegetables, owing to its restricted effect on the covalent bonds of low-molecular-mass compounds [10]. When it comes to the applications of HPP, the improved physiochemical and storage qualities of frozen albacore tuna [11], broccoli hummus [12], and multi-fruit smoothies [13] were reported. However, similar to other agricultural produce, bell peppers are highly susceptible to spoilage, especially after a series of fresh-cutting processes, and their quality tends to deteriorate. [14]. A previous study found that the storage of peppers under 5 kPa O_2_ + 5 kPa CO_2_ greatly reduced the ion leakage, controlled soft rotting, delayed softening, and maintained a lower metabolic activity of fresh-cut peppers [15]. Meanwhile, there has been little discussion about the application of HPP combined with N_2_ on the shelf-life of fresh-cut bell peppers.

Therefore, this study was conducted to evaluate the impact of the N_2_-assisted HPP treatment of fresh-cut bell peppers (FCBP) on the microorganisms, physicochemical properties, antioxidant capacity, and sensory quality after processing and during storage, to preserve the quality and enhance the shelf life of FCBP.

## 2. Materials and Methods

### 2.1. Chemicals

Folin–Ciocalteu, 2,2-diphenyl-1-picrylhydrazyl (DPPH), 2,4,6-tri-2-pyridyl-1,3,5-triazine (TPTZ), 7,8-tetramethyl-chroman-2-carboxylicacid (Trolox), and ascorbic acid were purchased from Sigma Aldrich (St. Louis, MO, USA). Guaiacol was supplied by Sinopharm Chemical Reagent (Shanghai, China). Acetonitrile of HPLC grade was purchased from Merck(Darmstadt, Germany). N_2_ (99.999%) gases were purchased from Beijing Beiwen Gas Co., Ltd. (Beijing, China). Other analytical-grade chemicals were provided by Beijing Chemicals Co., Ltd. (Beijing, China).

### 2.2. Preparation of FCBP

Red bell peppers *(Capsicum annuum* L.) at commercial maturity were purchased from a local market in Beijing (China) and washed for 3 min in distilled water. The cleaned peppers had their stems and seeds removed then were sliced into 3 cm × 3 cm with a ceramic knife and drained off for 3 min. A total of 200 g of pepper was packed in square plastic boxes (16 cm × 10 cm × 4 cm), covered with polyethylene (PE) film, then filled with 99.999% N_2_ using an atmosphere packaging machine (Dajiang Mechanical Equipment Co., Ltd., Wenzhou, China). The packed samples were temporarily stored at 4 °C until they were treated by HPP within 3 h. In the present study, both the untreated and HPP-treated samples were packed with N_2_ and the subsequent discussion will focus on the impact of different HPP treatments on the qualities of FCBP with the assistance of N_2_.

### 2.3. HPP Treatments

High-pressure processing was carried out with HPP equipment (30.0 L, Baotou Kefa Co., Ltd., Inner Mongolia, Baotou, China) at room temperature (25 ± 2 °C). The pressurization rate was about 120 MPa/min and the pressure was immediately released to 0.1 MPa (<3 s) after treatment. The pressure-transmitting fluid was distilled water. The treatment time did not include the pressure increase and the releasing time.

According to our pre-experiment, yeasts and molds (Y&M) were not detected in FCBP treated by 400 MPa/7.5 min (HPP-400) and 500 MPa/7.5 min (HPP-500) and the counts of total aerobic bacteria (TAB) were less than 3.00 log_10_ CFU/g, which also had a better performance in sensory evaluation compared to other HPP processing methods (300/400/500 MPa with 1/2.5/5/7.5 min, Table 1). Therefore, the samples were processed by HPP-400 and HPP-500 treatments in this study.

### 2.4. Storage Conditions

The untreated and HPP-treated samples were stored at 4 °C in the dark. Sample analyses were carried out at 0, 4, 8, 12, 16, 20, and 25 days of storage. After 8 days, the untreated samples were badly spoilt and not able to be analyzed for quality parameters.

### 2.5. Microbiological Analysis

When counting the viable microorganisms in samples, the total plate count method was applied [16]. A homogenizer bagmixer 400 (Interscience, Mourjou, France) was used to homogenize 25 g of samples and 225 mL of sterile normal saline (0.85% sodium chloride). The obtained pepper suspensions were serially diluted in sterile saline solution and plated in triplicate on nutritional agar for TAB counts at 37 °C for 48 ± 2 h and on the rose bengal medium for Y&M at 28 °C for 72 ± 2 h. After proper incubation, all the colonies were counted.

### 2.6. Physicochemical Characteristics Analysis

A GT6G7 juice extractor (Zhejiang Light Industry Machinery Co., Ltd., Zhejiang, China) was used to prepare FCBP pulp for pH, total soluble solids (TSSs), and weight loss ratio measurements.

The pH value was measured in triplicate at 25 ± 2 °C with a Thermo Orion 868 pH meter (Thermo Fisher Scientific, Inc., Waltham, MA, USA).

An Abbe refractometer (WAY-2S, Shanghai Precision and Scientific Instrument Co., Shanghai, China) was used to detect the total soluble solids (TSSs) at 25 ± 2 °C. The final results were reported as °Brix [16].

To analyze the weight loss ratio, the weights of untreated and HPP-treated samples were measured, respectively [17]. The weight loss ratio was calculated using Equation (1) as follows:

(1) $$\mathrm{Weight}\;\mathrm{loss}\;\mathrm{ratio}\;(\%)\;=\;\frac{m_{0}-m_{1}}{m_{0}}\times 100\%,$$

where m_0_ indicates the weight of untreated FCBP and m_1_ indicates the weight of HPP-treated samples during storage.

### 2.7. Color Assessment

The color assessment was evaluated at 25 ± 2 °C using a color measurement spectrophotometer (Hunter Lab Color Quest XE, Hunter Associates Laboratory, Inc., Reston, VA, USA) in the reflectance mode. The standard illuminant source inside the instrument (type of light source: D65) was used. Samples of crushed FCBP were loaded into a quartz cuvette (50 mm diameter), carefully removing air bubbles, and placed under the measuring aperture of the spectrophotometer [18]. The color of FCBP was expressed in L*, a*, and b* values. The total color difference ∆E is a parameter that describes the overall color difference of HPP-treated samples compared to the reference sample. It was calculated using Equation (2) as follows:

(2) $$\Delta E\;=\;\sqrt[{2}]{\left[{\left({\Delta L}^{*}\right)}^{2}+{\left({\Delta a}^{*}\right)}^{2}+{\left({\Delta b}^{*}\right)}^{2}\right]},$$

where ΔL* = (L*_1_ − L*_0_); Δa* = (a*_1_ − a*_0_); and Δb* = (b*_1_ − b*_0_). Subscript “0” indicates the color value for the reference sample (untreated FCBP at day 0) and subscript “1” indicates the color value for the sample being analyzed. All the measurements were conducted ten times, and the results were averaged.

### 2.8. Texture Profile Analysis

The texture profile analysis was valued by a texture analyzer (TX-XT Plus, Stable Micro System, Scarsdale, NY, USA) to evaluate the hardness of FCBP, following the method of Tangwongchai et al. [19] with some modifications. Each sample was cut into a 10 mm × 10 mm × 3 mm size for testing and was axially compressed two times to 30% of the original height with a 38 mm cylinder probe at a pretest speed of 1 mm/s, a test speed of 1 mm/s, and a post-test speed of 2 mm/s. Ten determinations were performed for each treatment. From the resulting force–time curves, the hardness (N) parameters were obtained and the average hardness values were calculated.

### 2.9. Determination of Total Phenols

The FCBP was ground and filtered, then the pepper pulp was centrifuged (GR21G, Hitachi Koki Co., Ltd., Tokyo, Japan) at 8000× *g* for 15 min at 4 °C. The supernatant was gathered and diluted 10 times with distilled water for further analysis.

Total phenols were evaluated according to Ryu and Koh [20] with slight modifications. A total of 0.1 mL of diluted sample was mixed with 2 mL of the Folin–Ciocalteu reagent (previously diluted 10-fold with distilled water). After being incubated for 5 min, 1.8 mL of 7.5% Na_2_CO_3_ (*m*/*v*) solution was added. After 1 h, the absorbance of the mixture was measured at 765 nm. (UV-726, Shimadzu, Shanghai, China). The results were expressed as the milligrams of gallic acid equivalent per 100 g of FCBP (mg GAE/100 g).

### 2.10. HPLC Analysis of Ascorbic Acid

For the extraction and analysis of ascorbic acid in FCBP, the method was proposed by Cao et al. [21] with modifications. A total of 2 g of the ground FCBP flesh was mixed with 4 mL of metaphosphoric acid (2.5%) and diluted to 10 mL using distilled water after filtration. After passing through a 0.45 μm cellulose nitrate membrane, the FCBP was ready for testing.

Ascorbic acid was separated and detected by a liquid chromatograph (LC-20AT) equipped with a UV-Vis detector (SPD-20AV) from Shimadzu Corporation (Kyoto, Japan). The separation was performed using Sunfire TM C18 from Waters (Milford, Massachusetts, USA). The mobile phase was an isocratic solvent system consisting of 95.00% monopotassium phosphate (50 mM, pH = 3.0) and 5.00% acetonitrile. The flow rate was 1.0 mL/min and aliquots of 20 μm were injected. The detection was performed in absorbance mode at 245 nm. Whole analyses were conducted at room temperature (25 ± 2 °C). A calibration curve was calculated using an external standard and used for quantification. Results were expressed as milligrams of ascorbic acid per 100 g of FCBP.

### 2.11. PPO Activity Assay

The polyphenol oxidase activity of the samples was analyzed according to Cao et al. [12], with some modifications. To extract the crude enzymes, 5 mL of FCBP flesh was blended with 25 mL of phosphate buffer solution (0.2 M, pH 6.5) and then centrifuged at 4000× *g* for 10 min at 4 °C. Crude enzymes were obtained from the supernatant after centrifugation. Subsequently, 0.5 mL of crude enzymes was thoroughly mixed with 3 mL of 1.0% o-methoxyphenol (diluted with 0.2 M, pH 6.5 phosphate buffer solution) and 10 μL of 1.5% hydrogen peroxide. The absorbance of the mixed solution was recorded every 10 s for 5 min at 470 nm (UV-726, Shimadzu, Shanghai, China). The enzyme activity unit (U) was defined as the change in absorbance of 0.001 units caused by 1 mL of enzyme extraction in 1 min.

### 2.12. Determination of Antioxidant Capacity

To study the antioxidant activity of FCBP, the free-radical scavenging effect on the ·DPPH radical and ferric reducing/antioxidant power (FRAP) was evaluated, following the method described by Gao et al. [22].

#### 2.12.1. DPPH Assay

At the beginning of the reaction, 100 μL of 10-fold diluted FCBP flesh was added to a cuvette containing 4 mL of a methanol solution (0.14 mol/L) of the methanolic ·DPPH solution. The mixture was kept in the dark for 50 min at room temperature and then its absorption was measured at 517 nm. Determinations were made using a UV-726 spectrophotometer (Shimadzu, Shanghai, China). Trolox solutions within the range of 100–1000 μM were used for calibration and the baseline was corrected by methanol.

#### 2.12.2. FRAP Assay

Freshly prepared FRAP solution contained 25 mL of 0.3 M acetate buffer (pH 3.6), 2.5 mL of 10 mM TPTZ (dissolved in 40 mM HCl), and 2.5 mL of 20 mM ferric chloride. A total of 4 mL of FRAP solution was mixed with 10-fold diluted pepper flesh in the dark at 37 °C for 10 min. The ferric reducing ability of the samples was measured by detecting the increase in absorbance at 593 nm with a UV-726 spectrophotometer (Shimadzu, Shanghai, China). Trolox solutions within the range of 100–1000 μM were used for calibration. The results were expressed as the radical scavenging activity of ·DPPH and FRAP, and were calculated by Equation (3) as follows:

(3) $$\mathrm{Radical}\;\mathrm{scavenging}\;\mathrm{activity}\;=\;\frac{A_{1}-A_{2}}{A_{1}}\times 100,$$

where A_1_ is the absorbance of the untreated sample at 517 or 593 nm and A_2_ is the absorbance in the presence of FCBP extract.

Antioxidant activity is expressed as millimoles of Trolox equivalents per kilogram of sample.

### 2.13. Sensory Evaluation

Ten volunteers (College of Food Science and Nutritional Engineering at the China Agricultural University, six woman and four man, aged 22–25) participated on the sensory test. HPP-treated and untreated samples were given to the participants, for a sensory evaluation on storage days 0, 4, 8, 12, 16, 20, and 25. To understand the characteristics of good-quality FCBP and the meaning of the different terminologies used in the sensory evaluation, all 10 participants had been trained for a sensory test at least once previously. Samples were evaluated for their sensory characteristics (taste, flavor, texture, and appearance) and overall acceptability on a 5-point scale (Table 2).

### 2.14. Statistical Analysis

All the experiments were carried out in triplicate and the average values were reported. The data were analyzed using statistical software (SPSS 17.0, Chicago, IL, USA). The results were expressed as mean ± S.D. Data were analyzed with one-way analysis of variance and Tukey multiple comparison tests (significance level *p* < 0.05) to verify whether mean values were significantly different.

## 3. Results and Discussion

### 3.1. Microbiological Analysis

The number of surviving cells after HPP treatments was determined by monitoring the TAB and Y&M counts. As shown in Table 3, the initial counts of TAB and Y&M in the untreated sample were 4.18 and 2.23 log_10_ CFU/g, respectively.

Both HPP-400 and HPP-500 treatments resulted in the reduction in Y&M to a level below the detection limit (10 CFU/g) during 25 days of storage at 4 °C. Similarly, several studies showed that Y&M were not detected immediately after HPP treatments, and survivors were kept below the detection limit in cupped strawberry [22] and banana puree [16].

Meanwhile, the counts of TAB in samples were significantly reduced to 2.69 and 2.15 log_10_ CFU/g after HPP-400 and HPP-500 treatments, respectively. The counts of TAB increased both in HPP-treated samples and untreated ones during storage. Meanwhile, the counts of TAB were consistently lower than 4 log_10_ CFU/g after the HPP-400 and HPP-500 treatments during storage, which showed that there was better microbiological stability after HPP treatment in comparison with untreated ones. This demonstrated that HPP is an effective technique for inactivating the microorganisms in FCBP. Similar results were found in fresh-cut cucumber slices [23] and precut lettuce [24]. With adiabatic compression and rapid expansion during HPP treatment, structural damage to the cell membranes of microorganisms occurs, and the inactivation of enzymes and the denaturation of active compounds, both of which are lethal to bacteria [10]. Furthermore, the presence of N_2_ in packed FCBP could also inhibit the proliferation of microorganisms during storage in the present study.

### 3.2. Chemical and Physical Analysis

The changes in the pH, TSS, and weight loss ratio of FCBP during storage at 4 °C are shown in Table 3. There was a significant change in the pH values after both HPP-400 and HPP-500 treatments. This might have been caused by the instantaneous leaching of acidic components after HPP treatment (e.g., phenolic compounds). Furthermore, the pH values were reduced generally in all FCBP during the 25-day refrigeration period, which may be attributed to the formation of organic acids produced by the microbial proliferation of FCBP.

The increase in TSS in FCBP was found after HPP treatment. Partially due to the textural damage of FCBP, the ingredients were concentrated relatively. Meanwhile, fluctuating TSS values were observed in both HPP-400 and HPP-500 treatments, which could have been caused by the dynamic balance between the loss of water molecules and the leaking of ruptured FCBP cells after HPP treatment. Whereas, Gallotta et al. [25] found that the TSS content of fresh-cut nectarines rose during 15-day storage with a reduced sample weight owning to the fruit ripening and the increasing production of ethylene. However, these effects may have been suppressed in the present study, as the packages contained 99.99% N_2_ and the after-ripening effect of fruit requires oxygen [26].

Due to the textural damage, the weight loss ratio of the FCBP increased substantially after the HPP treatment. Throughout the storage period, the rates of increase in the weight loss ratio after HPP-400 and HPP-500 treatments were 0.21% and 0.23%, respectively, and the weight loss ratio of HPP-500 tended to be stabilized for 12–25 days. There is no doubt that a series of processing operations, including HPP, could lead to the mechanical wounding of fresh-cut vegetable tissues [27], providing physical conditions for FCBP to lose weight after HPP-400 and HPP-500 treatments. Meanwhile, peppers are highly prone to lose water and corrupt naturally during long-term storage [28]. Presently, the HPP ensured the microbiological safety of FCBP, while a higher weight loss ratio was inevitably found after HPP treatment. Further studies need to focus on this undesirable result of FCBP.

### 3.3. Hardness Analysis

As shown in Table 3, the hardness of FCBP was 11.54–13.44 N after the HPP treatments, whereas the hardness of the untreated samples was 28.92 N. The texture loss observed in FCBP after HPP-400 and HPP-500 treatments could be defined as an instantaneous pressure softening. The leaching and non-enzymatic depolymerization of cell wall pectin in FCBP after the HPP application could be another reason for this effect [29,30]. For cherry tomatoes, Tangwongchai, Ledward, and Ames [19] found that the firmness was reduced by 90% after 400 MPa/20 min treatment. Similarly, strawberry halves were processed at 400–550 MPa for 0.1–20 min, reaching a maximum loss of 80% in hardness [31]. Moreover, the hardness of untreated samples decreased rapidly during 0–8 days of storage at 4 °C. However, none of the HPP-treated FCBP experienced a significant reduction in hardness over 25 days.

### 3.4. Color Analysis

The color of vegetables has a remarkable impact on consumer appreciation and acceptance. Processing had a significant effect on the color variables of FCBP in the present study (Table 4). There was no significant difference in the L* value between the HPP-treated and untreated samples at day 0. This result was in agreement with previous studies with fresh-cut peaches [32] and nectarines [33]. It has also been found that, regardless of the HPP treatment conditions, the chromatic parameters remained almost unaffected [32]. A lower L* value is an indicator of darkening and enzymatic browning, which is one of the factors most limiting the shelf life of fresh-cut products. Since HPP treatment could induce damage to the structure and the breaking of cell walls in fruits, it would facilitate enzyme (mainly PPO) and substrate contact, thus affecting the color of fruits. Meanwhile, N_2_-assisted HPP treatment, a technology that ensures the absence of oxygen inside packaging and inactivates PPO activity effectively, contributes to the preservation of L* by inhibiting enzymatic browning for 25 days.

There were significant (*p* < 0.05) effects of HPP treatment on the a* and b* values of FCBP. The HPP-treated samples became redder (higher a* values) and more yellow (higher b* values) at day 0. This result can probably be attributed to the cell disruption and release of pigment compounds after HPP treatment. Oliveira et al. [29] found a significant decrease in the a* and b* values in Peruvian carrot after both 600 MPa/5 min and 600 MPa/30 min. Meanwhile, significant increases in the a* and b* values of pawpaw pulp were found after 600 MPa/76 s [34]. The disparity between our observations and those reported from other studies may be attributed to differences in the HPP treatment conditions and the types of fruit and vegetables used. During storage for 25 days, HPP-treated samples showed a decrease in their a* and b* values. Thus, a color shift toward negative a* and negative b* directions indicated less red and less yellow in the samples, which was probably due to the significant degradation of chromogenic compounds, such as decreases in the amounts of carotenoids, flavonoids, and anthocyanins during storage.

The ∆E value, which is an indicator of total color difference, also showed that there were significant differences between untreated and treated samples. It has been considered that a ∆E of two would be a noticeable visual difference for a number of situations [35]. Thus, in this study noticeable changes were observed in the color of HPP-treated samples in comparison to that of untreated ones, which was probably due to the significant differences in the L*, a*, and b* values between the untreated and treated samples. During whole storage, the decrease in the ∆E of HPP-treated FCBP was related to the gradual degradation of chromogenic compounds, which diminished the color difference from the untreated sample. In contrast, the color behaviors of HPP-400-treated FCBP were more stable than those of HPP-500, as manifested by the lower ∆E and smaller changes in color values.

### 3.5. PPO Activity

**Figure 1:**
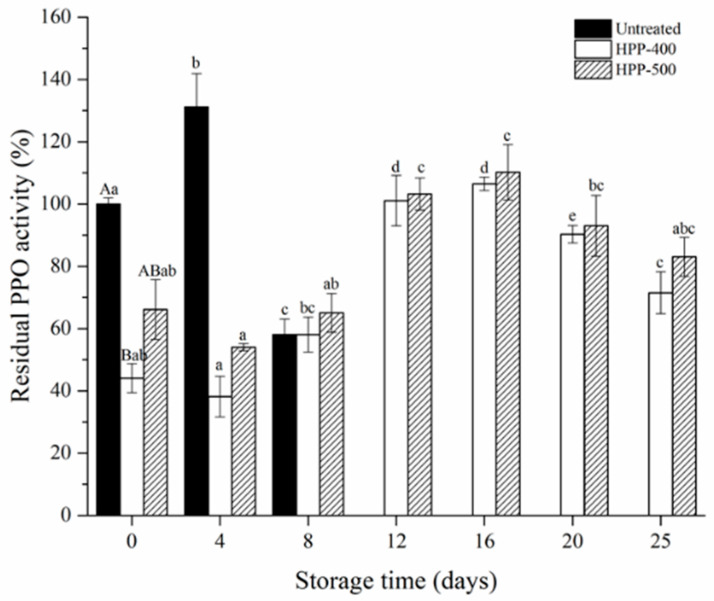
Changes in residual PPO activities of fresh-cut bell pepper during storage at 4 °C. The capital letters within one column indicate significant differences at day 0 (*p* < 0.05). Values with different letters within one column are significantly different during storage (*p* < 0.05).

Figure 1 shows the change in the residual PPO activity of FCBP during storage. After HPP-400 and HPP-500 treatments, the residual PPO activity in the FCBP was 44.09% and 66.13%, indicating that the PPO activity was passivated by the HHP treatment. Similarly, the application of HPP treatment has also been found to inhibit the PPO activity in fresh-cut potato [36] and fresh-cut peach [37].

During the first 8 days, HPP-treated FCBP showed lower PPO activity, whereas the PPO activity increased significantly after 8 days (*p* < 0.05). There was no significant difference in PPO activity between the HPP-400- and HPP-500-treated samples at day 25. The inactivation of PPO is highly dependent on several factors, especially the acidity of the medium, so the decrease in pH value in FCBP at the later stage of storage (8–25 days) was helpful for the recovery of PPO residual activity [38]. To maintain the color stability of FCBP during storage, additional hurdles should be considered to completely inactivate the residual activity of PPO in future studies.

### 3.6. Total Phenols and Ascorbic Acid

**Figure 2:**
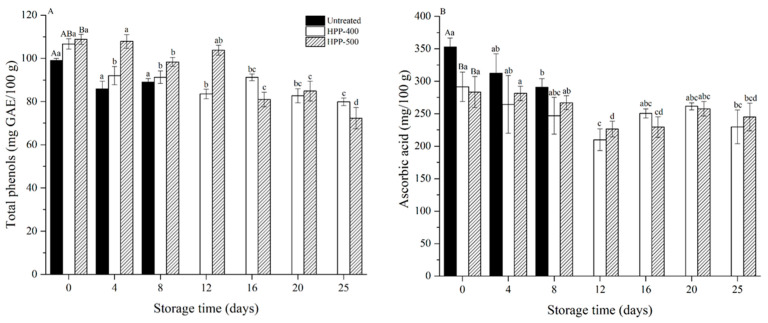
Changes in total phenols (**A**) and ascorbic acid contents (**B**) of fresh-cut bell pepper during storage at 4 °C. The capital letters within one column indicated significant differences at day 0 (*p* < 0.05). Values with different letters within one column are significantly different during storage (*p* < 0.05).

Changes in the total phenols and ascorbic acid contents of FCBP after HPP treatment during storage are shown in Figure 2. Compared to the total phenols content in an untreated sample (99.09 mg GAE/100 g), the total phenols in FCBP increased to 106.69 mg GAE/100 g and 108.80 mg GAE/100 g after treatments with HPP-400 and HPP-500, respectively. The previous study showed that HPP could cause a great compression of fruit microstructure and disruptions of the cell walls and membranes, leading to an increase in permeability and the leaking of total phenols in cells [38].

All the samples showed a significant reduction in their content of total phenols during storage (Figure 2A). After 25 days of storage, the losses of total phenols in the HPP-400- and HPP-500-treated FCBP were 19.44% and 27.19%, respectively. The leaching of phenolic compounds from flesh to juice was the main reason for this [22]. Although there was a high residual PPO activity, which was related to the degradation of phenolic compounds during storage [39], the presence of N_2_ inside the packaging did not provide proper conditions for the degradation of total phenols by PPO.

Figure 2B shows the changes in the ascorbic acid content of FCBP during storage. In all samples, the content of ascorbic acid in HPP-treated FCBP was significantly lower than that in untreated (*p* < 0.05). HPP treatment has a good performance in accelerating the extraction of ascorbic acid and other soluble compounds [40]. Therefore, in the present study due to the freely soluble characteristics of ascorbic acid, the decrease in ascorbic acid may be related to the drip loss of FCBP caused by HPP treatments. No difference in ascorbic acid content was observed between two HPP-treated samples.

The content of ascorbic acid decreased significantly in the untreated sample over 8 days (*p* < 0.05), yet no significant losses of ascorbic acid were found in the HPP-treated ones. At the end of storage, the ascorbic acid content decreased by 21.16% and 13.57% in the HPP-400- and HPP-500-treated FCBP, respectively. Drip loss, evidenced by the increasing weight loss ratio during storage, could be the main reason for this decrease. Furthermore, although FCBP was completely packaged with N_2_ instead of oxygen in this study, ascorbic acid can degrade anaerobically. Its influence factors include the presence or lack of oxygen, the amount of light, the presence of cupric ions, the temperature of processing, the storage time, and especially the pH [41]. The maximum degradation rate was reported at pH 4.0 and the minimum at pH 2.5–3.0 [42]. Therefore, it was reasonable to infer that after 8 days, with the pH decreasing to around 4.0 and the extension of storage time, the ascorbic acid degraded gradually in the HPP-treated samples.

### 3.7. Antioxidant Capacity Analysis

**Figure 3:**
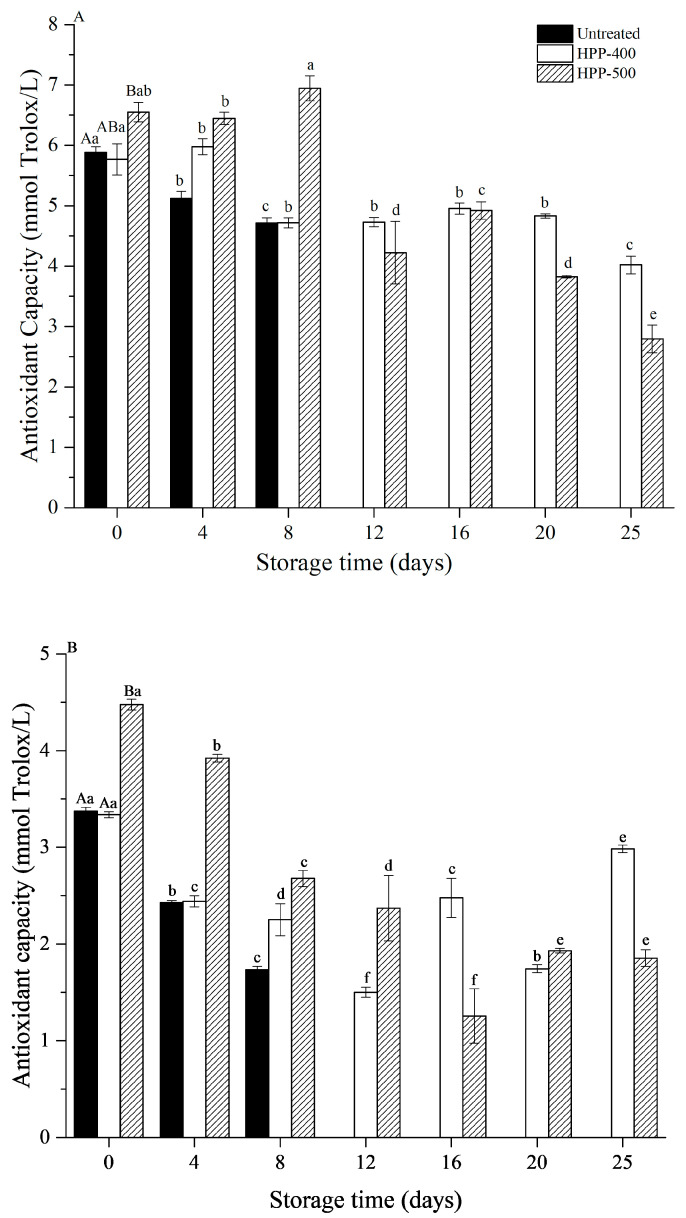
Changes in the antioxidant capacity (DPPH (**A**) and FRAP (**B**)) of fresh-cut bell pepper during storage at 4 °C. The capital letters within one column show significant differences at day 0 (*p* < 0.05). Values with different letters within one column are significantly different during storage (*p* < 0.05).

The antioxidant capacity in FCBP significantly increased after HPP-500 (*p* < 0.05), regardless of whether the DPPH or FRAP method was used (Figure 3). A similar result for antioxidant capacity was also found in pressurized swedes [43] and carrots [44].

In particular, N_2_-assisted HPP either increased or maintained the antioxidant capacity compared with untreated samples for 0–8 days, indicating that the N_2_-assisted HPP treatment was useful in preserving the antioxidant capacity of FCBP. Furthermore, the antioxidant capacity in HPP-500-treated samples was higher than that in HPP-400-treated ones for 0–8 days. However, HPP-400 treatment had a better performance in terms of the stability and persistence of antioxidant capacity in the second half of the storage period (12–25 days). After 25 days of storage, the DPPH antioxidant capacity decreased by 30.32% and 57.35% after treatments with HPP-400 and HPP-500, respectively. The FRAP antioxidant capacity decreased by 10.6% and 58.6% after 25 days, respectively. These results were probably due to the fact that a higher HPP treatment level (HPP-500) induced more severe structural damage to FCBP, leading to a better extracting effect of the antioxidant. Antioxidant capacity is closely related to the content of antioxidant substances (e.g., polyphenols and ascorbic acid) in samples. Therefore, the change in antioxidant capacity during storage can be explained by the change in antioxidant substances. During storage, the antioxidant capacity of the samples decreased significantly, which may be due to two reasons. One is that HPP treatment destroys the texture of the sample and that the weight loss ratio increases with the extension of storage time, resulting in the loss of polyphenols and other antioxidant substances; the other is that the rupture of the cells in samples allows the polyphenols to contact PPO, causing an enzymatic reaction, thus the polyphenols of the sample are consumed.

### 3.8. Sensory Evaluation

A sensory evaluation of the FCBP was carried out during 25 days of storage. The sensory scores were given by trained panelists (*N* = 10) according to a standard score sheet (Table 2) and they are shown in Table 5. The results showed that the sensory attributes of taste in the HPP-400-treated samples were similar to those for the untreated ones at day 0. Compared to HPP-400 treatment, the FCBP processed by HPP-500 received a higher score for the attributes of texture and appearance, probably because a better brightness (L* value) and lower weight loss ratio were found. Interestingly, a higher score for the attributes of taste and flavor were also found in the HPP-500-treated FCBP. Obviously, the HPP-500-treated samples had higher consumer acceptability. These results demonstrated that although there were significant changes in color, hardness, and other quality parameters, HPP processing did not negatively affect the sensory quality of FCBP.

The sensory scores of HPP-treated samples also decreased during storage, but the rate of decline was lower than in untreated ones, and it was not until day 25 that the samples began to corrupt. FCBP treated by HPP-500 performed better in terms of sensory quality than those treated by HPP-400 in the whole storage. The conclusion can be drawn that the sensory qualities of FCBP can be guaranteed after the application of N_2_-assisted HPP treatment. Furthermore, different processing conditions affected the sensory qualities of FCBP, both in terms of the storage stability and consumer acceptance of all sensory qualities.

## 4. Conclusions

N_2_-assisted HPP FCBP exhibited a high microbial reduction after processing and a better microbiological stability during storage. Color changes were noticeable between the untreated and treated samples after processing, and the ΔE values significantly decreased during storage. HPP effectively improved the total phenols content and antioxidant capacity of FCBP and significantly inactivated the PPO activity when compared to untreated samples. Besides this, HPP processing did not negatively impact the acceptability of all sensory attributes in contrast to untreated samples. However, due to the instantaneous pressure, softening was found in FCBP after HPP treatment, and significant weight loss ratios and losses of hardness were also found.

Therefore, N_2_-assisted HPP processing may be a good choice for the preservation of FCBP. Moreover, to further improve the quality and prolong the shelf-life of fresh-cut vegetables and decrease the damage to the natural structure after HPP, further research is required.

## Figures and Tables

**Table 1:** Sensory evaluation of fresh-cut peppers by high-pressure processing (HPP) treatment.

HPP Process		Color	Flavor	Texture	Appearance	Overall Acceptability
Pressure (MPa)	Holding Time (min)					
Untreated	-	4.20 ± 0.62a	3.90 ± 0.99a	4.20 ± 0.63d	4.40 ± 0.52d	4.50 ± 0.53c
300	1	3.78 ± 0.67a	4.11 ± 1.17a	3.44 ± 0.73cd	4.33 ± 0.71d	3.67 ± 0.71b
	2.5	3.78 ± 0.83a	3.22 ± 1.39	3.78 ± 0.67abc	3.56 ± 1.24abcd	3.22 ± 0.67ab
	5	3.44 ± 1.33a	3.00 ± 1.00a	2.56 ± 0.53ab	2.78 ± 1.20a	3.11 ± 0.78ab
	7.5	3.78 ± 0.67a	3.44 ± 0.88a	3.78 ± 0.67cd	4.00 ± 1.12bcd	3.50 ± 0.50ab
400	1	3.78 ± 0.83a	3. 33 ± 1.00a	3.22 ± 0.67bcd	4.11 ± 0.78cd	3.50 ± 0.50ab
	2.5	3.33 ± 1.12a	3.11 ± 1.17a	2.56 ± 0.53ab	3.11 ± 1.17abc	3.00 ± 0.50ab
	5	3.33 ± 1.23a	3.00 ± 1.32a	2.33 ± 0.50a	3.11 ± 1.17abc	2.78 ± 0.97a
	7.5	4.22 ± 0.67a	3.44 ± 0.88a	3.78 ± 0.97cd	4.33 ± 0.87d	3.72 ± 0.67b
500	1	4.11 ± 1.05a	3.22 ± 1.20a	3.33 ± 0.71cd	4.33 ± 0.87d	3.67 ± 0.50b
	2.5	3.33 ± 1.12a	3.00 ± 1.58a	2.33 ± 0.50a	3.00 ± 1.12ab	3.00 ± 0.71ab
	5	3.56 ± 1.10a	2.89 ± 1.05a	2.33 ± 0.87a	2.89 ± 1.17a	2.78 ± 0.67a
	7.5	3.44 ± 0.88a	2.89 ± 1.17a	3.00 ± 1.00abc	4.11 ± 0.78cd	2.83 ± 1.00a

All data were the mean ± S.D., *n* = 3. Values with different letters within one column are significantly different (*p* < 0.05). -, represents the pressure holding time of untreated groups was zero.

**Table 2:** The standard score sheet for the sensory evaluation of the fresh-cut bell pepper.

Score	Taste	Flavor	Texture	Appearance	Overall Acceptability
5	Refreshing, juicy and sweet; appropriate brittleness	Special pepper aroma; favorable soft and comfortable	Complete fruit tissue; stiff and springy	Full flesh; no drip loss	Excellent
4	Less sweet or juicy; a certain degree of brittleness	Special pepper aroma; relatively soft and comfortable	Certain springy	Full flesh; a little drip loss	Good
3	Lighter sweetness; general brittleness	Special pepper aroma	Slightly soft	Partly wrinkled; a little drip loss	General
2	No sweetness; tender	A little special pepper aroma	Soft	Partly wrinkled; serious drip loss	Bad
1	No sweetness; soft rotten	Pungent odor	Rotten	Sever wrinkled; serious drip loss	Unacceptable

**Table 3:** Changes in microorganisms, pH, total soluble solids (TSSs), weight loss ratio, and hardness of fresh-cut bell peppers during storage at 4 °C.

	Treatment	Storage Time (Days)						
		0	4	8	12	16	20	25
TAB (log_10_ CFU/g)	Untreated	4.18 ± 0.36Ca	5.21 ± 0.11b	6.91 ± 0.37c	---	---	---	---
	HPP-400	2.69 ± 0.07Ba	4.05 ± 0.17c	2.84 ± 0.50a	2.79 ± 0.13a	3.64 ± 0.18b	3.85 ± 0.35b	3.92 ± 0.11b
	HPP-500	2.15 ± 0.07Aa	2.35 ± 0.12a	3.80 ± 0.28b	1.69 ± 0.21c	2.73 ± 0.15c	3.61 ± 0.20b	3.83 ± 0.13b
Y&M (log_10_ CFU/g)	Untreated	2.33 ± 0.14a	2.52 ± 0.13a	3.18 ± 0.22b	---	---	---	---
	HPP-400	ND	ND	ND	ND	ND	ND	ND
	HPP-500	ND	ND	ND	ND	ND	ND	ND
pH	Untreated	5.09 ± 0.01Ba	5.08 ± 0.03b	5.05 ± 0.03b	---	---	---	---
	HPP-400	4.97 ± 0.02Aa	4.99 ± 0.01a	4.99 ± 0.03a	5.04 ± 0.27b	4.53 ± 0.33c	4.18± 0.03d	4.17± 0.04d
	HPP-500	4.99 ± 0.02Aa	4.99 ± 0.01a	4.98 ± 0.01a	5.00 ± 0.01a	5.03 ± 0.02b	4.99 ± 0.02a	4.45 ± 0.02c
TSS (^o^Brix)	Untreated	6.80 ± 0.17Aa	6.50 ± 0.17a	6.60 ± 0.10a	---	---	---	---
	HPP-400	7.27 ± 0.12ABab	7.83 ± 0.12c	7.70± 0.10bc	7.73± 0.15bc	7.80 ± 0.26c	7.77 ± 0.25c	7.00 ± 0.17a
	HPP-500	7.33 ± 0.12Babc	7.83 ± 0.21c	7.47± 0.12abc	7.73± 0.31bc	7.27 ± 0.15ab	7.60 ± 0.20bc	7.07 ± 0.12a
Weight loss ratio (%)	Untreated	0	1.1	4.3	---	---	---	---
	HPP-400	21.9	22.7	22.8	25.7	25.9	26.3	27.2
	HPP-500	18.7	23.1	23.5	24.4	24.2	24.4	24.5
Hardness (N)	Untreated	28.92 ± 1.22Ba	28.05 ± 1.28ab	21.31± 1.17b	---	---	---	---
	HPP-400	13.44 ± 1.45ABa	10.59 ± 0.80bc	11.09 ± 0.37bc	11.76 ± 0.22bc	10.88 ± 0.57bc	10.51 ± 0.33bc	9.65 ± 0.26c
	HPP-500	11.54 ± 0.93Aa	10.99 ± 0.45a	10.58 ± 0.78a	11.11 ± 0.28a	9.79 ± 0.24a	10.18 ± 0.29a	10.72 ± 1.95a

---, not tested; ND, not detected (detection limit <10 CFU/g); TAB, total aerobic bacteria; Y&M, yeasts and molds. All data were the mean ± S.D., *n* = 3. Values with different letters within one row are significantly different (*p* < 0.05). The capital letters within one column are significantly different at day 0 (*p* < 0.05).

**Table 4:** Changes in the color parameters of fresh-cut bell pepper during storage at 4 °C.

Process	Storage (Days)	L*	a*	b*	ΔE
Untreated	0	32.90 ± 2.53Aa	28.84 ± 4.47Aa	17.45 ± 3.89Aa	0
	4	30.93 ± 3.79a	28.67 ± 3.99a	20.04 ± 3.57ab	3.26
	8	31.52 ± 3.65a	33.67 ± 5.41b	26.56 ± 10.93b	10.40
HPP-400	0	31.06 ± 2.31Aa	36.38 ± 4.27Ba	27.94 ± 7.85Ba	13.05
	4	29.04 ± 3.13a	35.49 ± 6.52ab	30.30 ± 11.63a	14.97
	8	29.68 ± 2.01a	30.10 ± 4.33cd	22.15 ± 5.05ab	5.83
	12	32.00 ± 1.90a	27.70 ± 2.15cd	15.26 ± 2.35b	2.63
	16	30.01 ± 4.50a	30.50 ± 5.05bc	20.50 ± 6.36ab	4.52
	20	30.65 ± 8.613a	31.22 ± 8.79abc	21.78 ± 12.18ab	5.43
	25	30.94 ± 2.90a	25.13 ± 3.33d	14.53 ± 3.03ab	5.11
HPP-500	0	33.49 ± 1.37ABa	34.18 ± 6.54Ba	28.31 ± 14.93Bab	12.12
	4	30.08 ± 2.53ab	36.31 ± 6.69a	30.89 ± 8.42a	15.63
	8	27.83 ± 3.45b	30.75 ± 5.36b	24.16 ± 10.26bc	8.62
	12	28.01 ± 6.70b	26.82 ± 3.80b	16.76 ± 2.21c	5.34
	16	27.52 ± 3.57b	29.61 ± 4.12b	21.71 ± 5.19bc	6.91
	20	27.90 ± 3.76b	26.33 ± 2.06b	17.29 ± 3.85c	5.60
	25	31.62 ± 3.52ab	25.91 ± 4.31b	16.91 ± 6.15c	3.24

All the data were the mean ± S.D., *n* = 3. The capital letters within one column show significant differences at day 0 (*p* < 0.05). Values with different letters within one column are significantly different (*p* < 0.05).

**Table 5:** Variations in the sensory scores of fresh-cut bell pepper during storage at 4 °C.

Process	Storage (Days)	Taste	Flavor	Texture	Appearance	Overall Acceptability
Untreated	0	3.78 ± 0.67Aa	4.33 ± 0.86Aa	3.44 ± 0.73Aa	4.33 ± 0.50Aa	3.67 ± 0.61Aa
	4	3.78 ± 0.83a	3.67 ± 1.22a	3.33 ± 0.56a	3.55 ± 1.24a	3.22 ± 0.64ab
	8	2.00 ± 0.71b	1.67 ± 0.71b	3.40 ± 0.73a	3.44 ± 0.72a	2.78 ± 0.76b
HPP-400	0	3.75 ± 0.89Aa	3.50 ± 1.20BCa	2.75 ± 0.70Bab	3.88 ± 0.83Ba	3.38 ± 0.52Ba
	4	3.67 ± 1.12ab	3.22 ± 0.83a	3.03 ± 0.86ab	3.33 ± 0.70a	3.11 ± 0.78a
	8	3.78 ± 0.67a	3.44 ± 0.88a	2.77 ± 0.66a	4.00 ± 1.11a	3.50 ± 0.56a
	12	3.33 ± 1.12ab	3.11 ± 1.17a	2.55 ± 0.53ab	3.11 ± 1.17a	3.00 ± 0.51a
	16	2.78 ± 0.97ab	2.44 ± 0.73a	2.30 ± 0.50a	3.10 ± 1.17a	2.78 ± 0.97a
	20	2.78 ± 0.67ab	2.33 ± 0.50a	2.33 ± 0.50a	3.00 ± 1.12a	3.00 ± 0.71a
	25	2.33 ± 1.00b	2.44 ± 0.73a	2.33 ± 0.50a	3.11 ± 1.17a	2.78 ± 0.91a
HPP-500	0	4.20 ± 0.63Ba	3.90 ± 0.99ABa	3.40 ± 0.97Aab	4.40 ± 0.51Aa	3.50 ± 0.53Aab
	4	4.22 ± 0.83a	3.22 ± 1.20a	3.30 ± 0.70ab	4.33 ± 0.85a	3.67 ± 0.40ab
	8	4.22 ± 0.67a	3.40 ± 0.88a	3.70 ± 0.90a	4.33 ± 0.87a	3.72 ± 0.70ab
	12	3.56 ± 0.73abc	3.33 ± 1.00a	3.22 ± 0.67abc	4.11 ± 0.78ab	3.50 ± 0.50bc
	16	3.78 ± 0.67ab	3.22 ± 0.97a	3.50 ± 0.52ab	4.00 ± 1.12ab	3.50 ± 0.53bc
	20	2.78 ± 0.83bc	2.56 ± 0.88a	2.56 ± 0.50bc	3.00 ± 1.00b	3.11 ± 0.78bc
	25	2.63 ± 0.74c	2.50 ± 0.93a	2.25 ± 0.88c	3.00 ± 1.20b	2.75 ± 0.71c

All the data were the mean ± S.D., *n* = 3. The capital letters within one column show significant differences at day 0 (*p* < 0.05). Values with different letters within one column are significantly different (*p* < 0.05).
